# Describing the Access Network by means of Router Buffer Modelling: A New Methodology

**DOI:** 10.1155/2014/238682

**Published:** 2014-07-24

**Authors:** Luis Sequeira, Julián Fernández-Navajas, Jose Saldana, José Ramón Gállego, María Canales

**Affiliations:** Communications Technology Group (GTC), Aragón Institute of Engeneering Research (I3A), Department of IEC, EINA, University of Zaragoza, Ada Byron Building, 50018 Zaragoza, Spain

## Abstract

The behaviour of the routers' buffer may affect the quality of service (QoS) of network services under certain conditions, since it may modify some traffic characteristics, as delay or jitter, and may also drop packets. As a consequence, the characterization of the buffer is interesting, especially when multimedia flows are transmitted and even more if they transport information with real-time requirements. This work presents a new methodology with the aim of determining the technical and functional characteristics of real buffers (i.e., behaviour, size, limits, and input and output rate) of a network path. It permits the characterization of intermediate buffers of different devices in a network path across the Internet.

## 1. Introduction

Traditionally, the available bandwidth, delay, and jitter between two end-to-end devices have been used as a parameter that can give a rough idea of the expected quality of service (QoS). But nowadays, we know that QoS is also affected by the behaviour of the intermediate router buffer, which is mainly determined by its size and its management policies. So, the buffer may cause different packet loss behaviour and may also modify some QoS parameters.

Many multimedia applications and services (e.g., videoconferencing, video streaming, peer-to-peer, and VoIP services) take advantage of various available bandwidth estimations techniques and tools (ABETT) in order to improve some QoS parameters. But all these techniques have one thing in common: they are focused on links estimations at the core network where buffer behaviour and its parameters are not the principal priorities.

In general, buffers are used as a traffic regulation mechanism in network devices. Current core routers make extensive use of AQM (active queue management) disciplines which are able to maintain a shorter queue length than drop-tail queues; this fights against bufferbloat and reduces latency. But these techniques (e.g., RED and SRED) require careful tuning of their parameters in order to provide good performance [1]. There exist QoS scheduling algorithms as weighted fair queuing (WFQ) which is a data packet scheduling technique allowing different scheduling priorities to statistically multiplexed data flows.

However, some network points may become critical bottlenecks, mainly in access networks, because these networks' capabilities are lower than the ones available in the backbone, being the main cause of packet loss of the discarding of packets in router queues. Mid- and low-end routers, which do not implement advanced traffic management mechanisms, are usually used in access networks. In this scenario, SME (small and medium enterprises) environments may be principally affected because of their modest infrastructure. So the design characteristics of router buffers and the implemented scheduling policies are of primary importance in order to ensure the correct delivery of the traffic of different applications and services, so it will be useful to include buffer parameters in the link capacity estimation.

On the other hand, it is true that the performance of TCP (transmission control protocol) has been extensively studied and a big number of variants ( SACK, New Reno, Vegas, etc.) have been deployed in order to improve it. Nevertheless, many multimedia applications and real-time services transport their information under UDP (user datagram protocol). So, the applications have to describe certain network behaviour for optimizing traffic.

Hence, the characterization of the technical and functional parameters of router buffer in SME environments becomes critical when planning a network or trying to provide certain levels of QoS. As a consequence, if the size and the behaviour of the buffer are known, some techniques can be used so as to improve link utilization, for example, multiplexing a number of small packets into a big one, fragmentation, smoothing traffic, and so forth.

A new methodology is presented in this paper in order to describe the access network by means of router buffer modelling (e.g., behaviour, size, limits, and input and output rate) by the use of four simple steps which will be detailed in Section 3.3. In particular we will estimate bandwidth, size, and behaviour because this gives us more useful link information, than only an estimation with ABETT techniques. In addition, this methodology has been deployed in order to solve problems of resource consumption for processing data and inaccuracies when obtaining input rate estimations and buffer size as well.

The paper is organized as follows: Section 2 presents the related work, and Section 3 describes the test methodology. The next section covers the experimental results, and the paper ends with the conclusions.

## 2. Related Work

### 2.1. Bandwidth Estimations

There exist several estimation techniques for obtaining available bandwidth. A performance evaluation of Pathload, Pathchirp, Spruce, IGI, and Abing in a flexible test bed was presented in [2]. The results demonstrate that ABETTs are far from being ready to be applied in all applications and scenarios. The evaluation includes scenarios in which the packet loss rate and the propagation delays of the links have been varied. Also, the amount of cross-traffic, the capacity of the links, and the cross-traffic packet size were tested for different values.

In [3], the authors define probabilistic available bandwidth as the largest input rate at which we can send a traffic flow along a path while achieving, with specified probability, an output rate that is almost as large as the input rate. The method is expressed directly in terms of the measurable output rate and includes adjustable parameters that allow the user to adapt to different application requirements. It was proposed as a new definition for available bandwidth and a novel framework that addresses some issues: provide a confidence interval, be suited to the task of multipath estimation in large-scale networks, and provide enough flexibility in terms of accuracy, overhead, latency, and reliability to adapt to the requirements of various applications.

Moreover, in [4] a novel tool for end-to-end ABETT PRM (probe gap models) called quality monitoring and estimation (QMoEs) was proposed, as a key component embedded in the RUBENS (rethinking the usage of broadband access for experience-optimized networks and services) architecture. The results indicate that QMoEs present a good performance in terms of accuracy and estimation time which validates the ABETT within the RUBENS framework.

### 2.2. Buffer Issues

Buffers are used to reduce packet loss by absorbing transient bursts of traffic when routers cannot forward them at that moment. They are instrumental in keeping output links fully utilised during congestion times.

For many years, researchers accepted the so-called* rule of thumb* (or bandwidth delay product, BDP) to obtain the amount of buffering needed at a router's output interface. This rule was proposed in [5] and it is given by *B* = *C* × RTT, where *B* is the buffer size, RTT is the average round-trip time, and *C* the capacity of the router's network interface. In [6] a reduced buffer size was proposed by dividing the BDP by the square root of the number of TCP flows, *N*, $B=C\;\times \;\text{RTT}/\sqrt{N}$. This model was called* small buffer*. In [7] the use of even smaller buffers, called* tiny buffers*, was suggested considering a size of some tens of packets.

In [8], the authors presented a simple algorithm to manage a drop-tail queue, which adapts its size based on traffic conditions in order to obtain a minimum size and providing high level of utilization. The results show that adaptive drop-tail achieves significantly smaller queues than current approaches at the expense of 1-2% of the link utilization.

The buffer can be measured in different ways: maximum number of packets, amount of bytes, or even queueing time limit [9, 10]. For example, in [11] the routers of two manufacturers were compared, and it was observed that one measures the buffer in packets, whereas the other one does it in milliseconds.

Moreover, the buffer must play an important role when planning a network because it can influence the packet loss of different services and applications. The reason for this is that there is a relationship between router buffer size and link utilization, since an excessive amount of memory would generate a significant latency increment when the buffer is full. On the other hand, a very small amount of memory in the buffer will increase packet loss in congestion time. As a consequence, the knowledge of the buffer behaviour is an interesting parameter which can be considered when trying to improve link utilization.

### 2.3. The Influence of the Buffer on Real-Time Services

The influence of the router buffer on the subjective quality experienced by users of a interactive service (i.e., an online game) with very tight real-time requirements was studied in [4], showing the mutual relationship between the size and policies of the buffer and the obtained subjective quality, which mainly depends on delay and jitter in this case.

The study has been conducted, showing that* tiny buffers* are more adequate in order to maintain game quality in acceptable levels. Since, if it is too big it may add delay and jitter which are not acceptable for gamers.

A popular online, multiplayer, game server was studied in [12]; the paper shows the results of a 500-million packet trace in which the traffic behaviour describes a loaded game server and can be attributed to the fact that current game designs target the saturation of the narrowest, last-mile link. Online games try to provide relatively uniform experiences between all players, maximizing the interactivity of the game, so they fix their usage requirements in such a way as to saturate the network link of their lowest speed players.

The authors also comment that facing the stringent demands on interactivity, routers must be designed with enough capacity to manage such bursts without delay. But current routers are designed for bulk data transfers with larger packets, so a significant deployment of online game servers will have the potential for overwhelming the current networking equipments.

Several studies have characterized P2P video streaming applications and have measured their impact in the communication networks traffic. So, in [13] a traffic characterization of major P2P-TV application was presented and they concluded that the traffic consists in a mixture of small packets (signalling packets) and large packets (video packets). In addition, the generation of high rates of small packets may penalize the video packets and consequently the peer's behaviour within a P2P structure may not be as expected. The application relies on UDP traffic and it is revealed that this application faces a high overhead; about 60% of packets correspond to signalling, and the other 40% correspond to video data packets.

**Figure 1:**
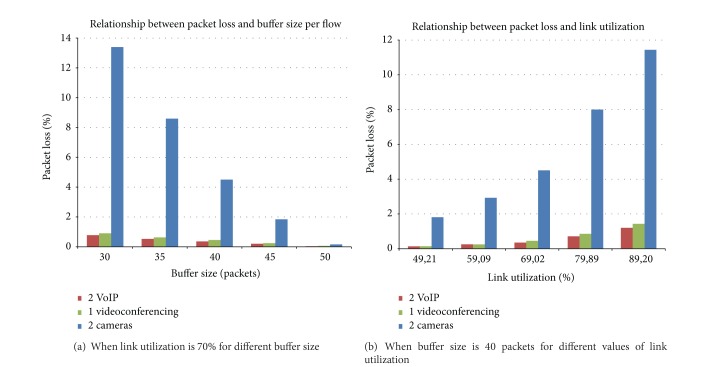
Packet loss.

In [14], the results show that the presence of a bursty application (video surveillance) causes packet loss for all the coexisting applications, even for those generating constant bit rate traffic (VoIP). In addition, packet loss decreases when buffer size is increased, because big buffers can absorb the burst produced by the traffic mix. As expected, packet loss increases when link utilization grows in the case of 40-packet buffer (Figure 1).

The tests were deployed in a scenario in which two IP camera flows, one videoconferencing session, and two VoIP calls are used as test traffic in two different tests: in the first one, the Internet access link was set to an average link utilization of 70% and different values of the buffer size were tested. In the second tests, the buffer size of the Internet access router was fixed at 40 packets and different values of the access bandwidth were used, so consequently different levels of link utilization ranged from 50% to 90%.

## 3. Methodology for the Characterization of Internet Paths

**Figure 2:**
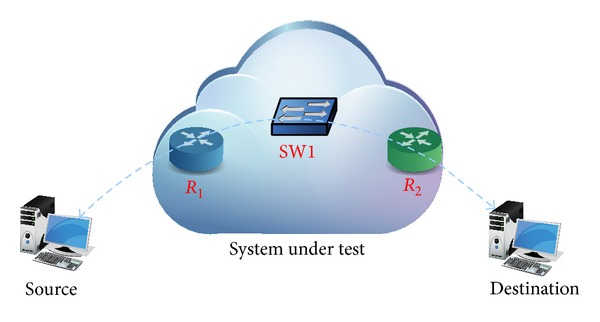
Network path and topology used for tests.

The network path (Figure 2) is uncertain for most applications and services which only measure the available bandwidth, in order to limit the generated traffic and rarely to attune it (e.g., trough smoothing it). Thus, we propose a method for applications to discover and get advantage of knowing network characteristics by means of finding the buffer behaviour models which may be useful to correctly attune the traffic.

### 3.1. Network Path Model

**Figure 3:**
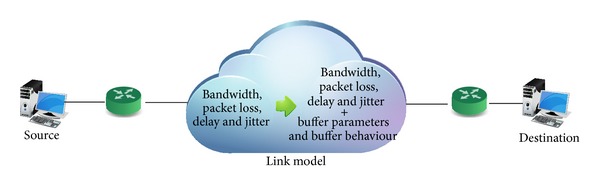
Link model parameters.

Traditionally a network path can be characterized by bandwidth, packet loss, and delay. The premises of this work are that most of the network characteristics can be explained by buffer models. We recommend a characterization by including buffer parameters (size and input and output rate) and buffer behaviour; see Figure 3.

**Figure 4:**
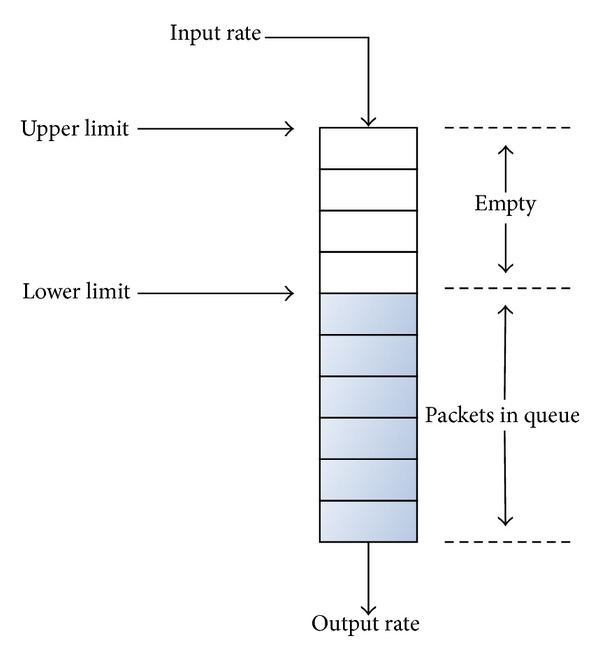
A particular buffer behaviour.

In this work, the buffer model only considers FIFO queues since they are the most common in real access network devices as it was observed in [15]. The same work also reported that the behaviour of these buffers can be characterized as follows: once the buffer gets completely full, no more packets or bytes are accepted until a certain amount of memory is available; see Figure 4. Thus, an* upper limit* and a* lower limit* can be defined: when the* upper limit* is reached, no more packets or bytes are accepted until the size of the buffer corresponds to the* lower limit*. There are some cases in which the difference between* lower limit* and* upper limit* is as small as one packet; however, this difference sometimes reaches tens of packets. Generally speaking, a buffer drops packets in bursts. The number of packets per burst depends on the relationship between the input and output rates. When these rates are very similar, the number of packets in the burst can be just one packet.

### 3.2. Test Procedure

The scheme of the tests is shown in Figure 2. There is a “system under test” (SUT from now), which may be either a single device or an entire network. The test is based on sending a burst of UDP packets, all with the same length, from the source to the destination machine, so as to produce a buffer overflow in the SUT. All the transmitted packets are identified by a sequence number included in the payload.

### 3.3. Methodology

**Figure 5:**
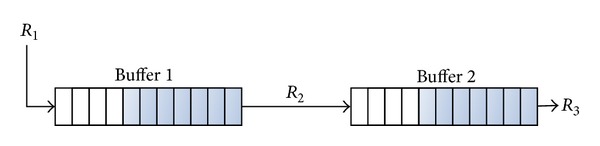
Two concatenated buffers.

The methodology is based on the premise that the output rate can be obtained from traffic capture at the destination device. This output rate depends on the technology used in each case (Ethernet, Wi-Fi, Cablemodem, and others). Different buffers can be detected by means of a packet loss pattern analysis when more than one bottleneck is in the path. In most cases, one buffer is the main cause of packet loss in a network path and sometimes two buffers can get into overflow at the same time in an access network; for this reason, we will use as an example the case of two concatenated buffers to present this methodology (Figure 5).

**Figure 6:**
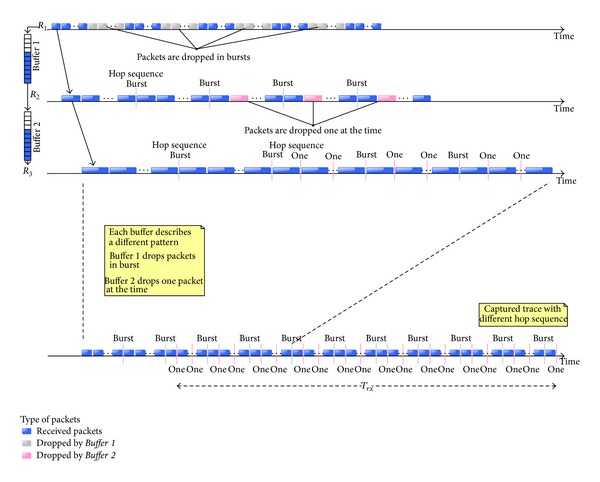
Dropped packets for two concatenated buffers.

The output rate can be easily determined by the destination trace because we know all arrived packets and the time of each one; see Figure 6. Also, the input rate can be estimated counting the dropped packets in the destination trace by means of the sequence number included in the payload.

**Figure 7:**
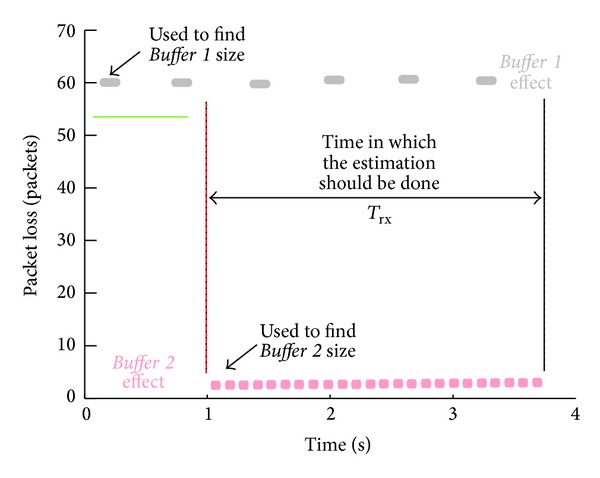
Packet loss map for two concatenated buffers.

A packet loss map is useful not only to determine the amount of losses but also to observe the packet loss patterns which may define each buffer model. It is a simple packet loss scatter (see Figure 7). We should note that the information on this map is equivalent to the buffer occupancy under the same conditions, also a simple way to see the packet loss patterns (hop sequence) which are difficult to deduce from Figure 6.

The methodology consists of four simple steps which will be described as follows:

(1) Methodology{⟹Analyze  packet  loss  patterns⟹Determine  rates⟹Infer  locations⟹Estimate  buffer  size.

#### 3.3.1. Analyze Packet Loss Patterns

The analysis consists of determining the number of congested buffers and the packet loss rate of each one. As an example, in Figure 7 we can see two different patterns which correspond to two buffers. The packet loss for the first is 60 packets per burst, while the second only drops 1 packet per burst. Also, we can find the number of dropped packets in a given period of time *T*_*rx*_ for* Buffer *1 (*N*_1_) and* Buffer *2 (*N*_2_).

#### 3.3.2. Determine Rates

The output rate (*R*_3_ in Figure 6) can be calculated with (2), from the destination trace because we know all the arrived packets, *N*, and the arrival time of each one; see Figure 6. The input rate (*R*_1_ in Figure 6) is obtained with the arrived packets and all the dropped ones (3). We must estimate the rates in the period in which we observed all the packet loss patterns *T*_*rx*_ (see Figures 6 and 7) since it has been observed that it is the most stable period in terms of output rate and packet loss. Consider

(2) $$\begin{array}{c} R_{3}=\frac{P_{L}}{T_{R_{x}}}\times N, \end{array}$$

(3) $$\begin{array}{c} R_{1}=\frac{P_{L}}{T_{R_{x}}}\times \left(N+N_{1}+N_{2}\right), \end{array}$$

where *P*_*L*_ is the* packet length*.

If we obtain several packet loss patterns (see Figure 6), we can also calculate intermediate rates. When there are two buffers in the path a general equation (4) can be defined in which two values can be found. The intermediate rate calculation (*R*_2_ in Figure 6) will generate two different possible values. In order to obtain the correct value, a new test with a new generated rate between these two value rates is needed. If the results show (by observing packet loss maps) that both buffers are overflowed then the correct value is the lowest one; otherwise it is the highest. Consider

(4) $$\begin{array}{c} R_{2}=\{\begin{array}{c} \frac{P_{L}}{T_{R_{x}}}\times \left(N+N_{1}\right)\\ \frac{P_{L}}{T_{R_{x}}}\times \left(N+N_{2}\right). \end{array} \end{array}$$

#### 3.3.3. Infer Locations

The packet loss rate is defined by the relationship between input and output rates of any buffer. So, when the packet loss rate of each buffer is known, we can compare the rate with the relationship of the input and output rates (*R*_1_, *R*_2_, and *R*_3_) to obtain the buffer position; see Figure 6.

#### 3.3.4. Estimate Buffer Size

**Figure 8:**
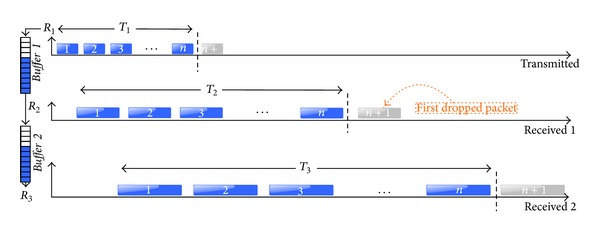
Estimating buffer size, from the last received packet before first packet loss.

When we know the buffer input and output rates, then buffer size can be estimated if we find the latency of a packet in the buffer when it is full. In this case, we use the last received packet before the first packet loss, as it is shown in Figure 8, because this is the packet which completely fills the buffer. In addition the sequence number (SN) of this packet *n* can give us the number of packets sent in certain time *T*. The *n*th packet gets into the* Buffer*_*m*_ in a time: *T*_*m*_ = *n*∗*P*_*L*_/*R*_*m*_, and the output time is *T*_*m*+1_ = *n*∗*P*_*L*_/*R*_*m*+1_. The packet latency is *T*_*m*+1_ − *T*_*m*_, so the buffer size, in packets, of the *m* buffer can be estimated using (5), which only depends on the rates relationship and the *n* arrived packets before the packet loss in each buffer.

The number of arrived packets to a certain buffer, before the first packet loss, depends on which buffer drops packets first and the physical location of the buffer (see Figure 6) described above. From Figure 6 we know that* Buffer *1 is physically before* Buffer *2, so the dropped packets by* Buffer *1 will never arrive to* Buffer *2. From Figure 7 we know that* Buffer *1 is the first one in dropping packets, so we can deduce that *n*_1_ = SN_1_ and *n*_2_ = SN_2_ − *N*_1_. The same analysis can be applied when* Buffer *2 is before* Buffer *1. In this case the dropped packets by* Buffer *2 successfully pass through* Buffer *1, so *n*_1_ = *S*N_1_ and *n*_2_ = SN_2_. Consider

(5) sizeBufferm=(Tm+1−Tm)×Rm+1PL=nm×(1−Rm+1Rm).

With the aim of determining if the buffer is measured in number of bytes or packets, a new test should be done; in this case, the new test burst of UDP packets should use a different packet length. Now, we can calculate buffer size for all tests and compare the results: if the buffer size is the same for all tests, the buffer is measured in number of packets if not in bytes.

## 4. Experimental Results

**Figure 9:**
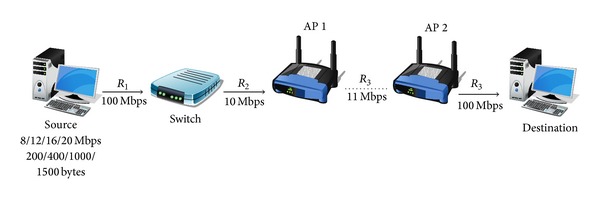
Topology used for estimating the buffer size in wired and wireless network.

Real tests have been deployed in a testbed and results are analyzed according to the procedures cited above. We have implemented a controlled network environment in order not only to reproduce the scenario in Figure 5 but also to study two different devices: a switch (3COM) and a Wi-Fi access point (Linksys WAP54G). The topology is shown in Figure 9.

The wireless link capacity is set to 11 Mbps. Packets of different sizes (200, 400, 1000, 1500 bytes) are used to determine if the buffer is measured in number of packets or in bytes. The presented results are the most significant.

### 4.1. Characterizing the Path

#### 4.1.1. Analyze Packet Loss Patterns

**Figure 10:**
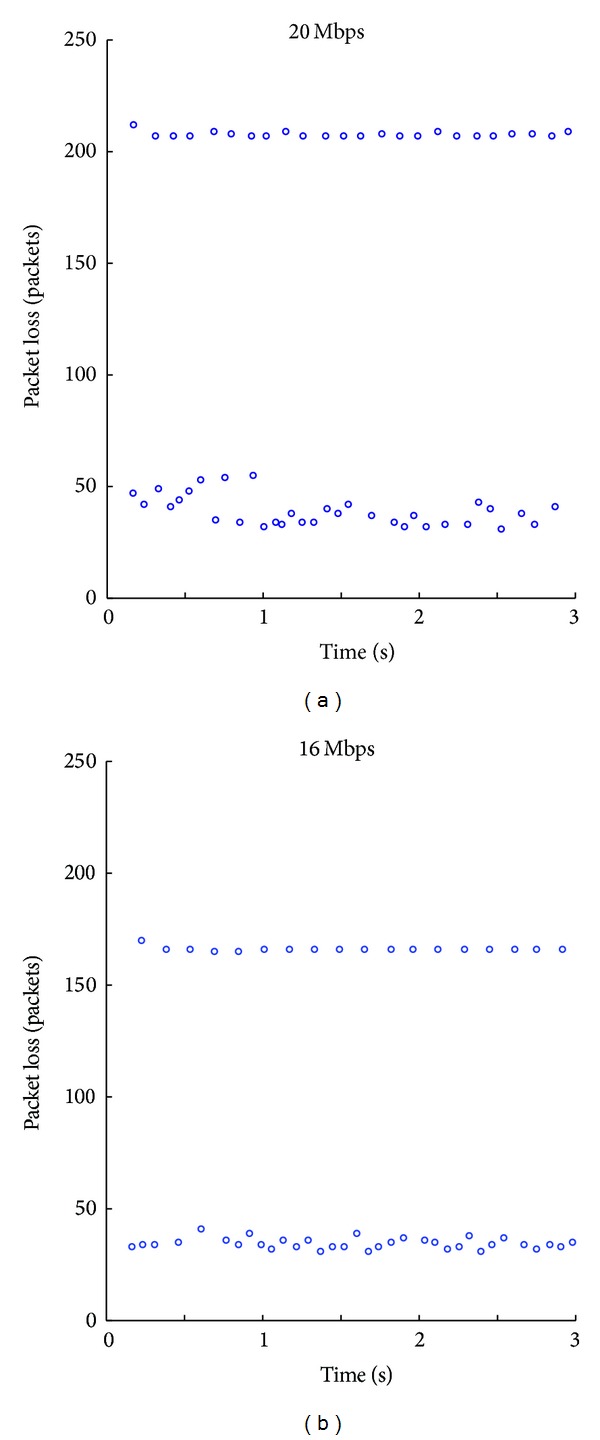
Packet loss patterns in the switch and the access point for different bandwidth amounts when packet size is 1500 bytes.

In order to obtain a reliable packet-loss map that permits the pattern analysis and be the least intrusive as possible, we generated a traffic of 20 Mbps with a packet length of 1500 bytes, which is considerably bigger than link capacity and it is not so intrusive as the interface maximum output rate.  Figure 10(a) shows two different packet loss patterns which can be determined by observing the groups of packets around the same packet loss value. The first pattern is roughly 45 packets and the second one 205. The second pattern is very constant while the first presents some dispersion.

#### 4.1.2. Determine Rates

We obtain output and input rates using (2) and (3) in the stable period; however output rate presents more variations due to Wi-Fi behaviour. The results are *R*_3_ = 6.5 Mbps (average) and *R*_1_ = 20 Mbps.  Figure 10(a) shows two patterns, so there is an intermediate rate *R*_2_. From (4) we obtain two possible values: *R*_2*a*_ = 18 Mbps and *R*_2*b*_ = 10 Mbps. In order to choose the correct value a new test is deployed with a rate of 16 Mbps (a value between *R*_2*a*_ and *R*_2*b*_). The results can be seen in Figure 10(b), which shows that two packet loss patterns appear. This means that both buffers get into overflow (the first pattern still maintains its value in 45 packets, but the second one has decreased to 160); thus the intermediate rate is 10 Mbps.

#### 4.1.3. Infer Locations

If we consider the buffer order as shown in Figure 6 and the obtained results (*R*_1_ = 20 Mbps, *R*_2_ = 10 Mbps, and *R*_3_ = 6.5 Mbps),* Buffer *1 must lose half the arrived packets, but* Buffer *2 only 35%. We compare these results with each packet loss pattern and we infer that* Buffer *1 drops bursts of 205 packets and* Buffer *2 drops bursts of 45. Furthermore,* Buffer *2 may correspond to the Wi-Fi access point buffer because it has more variations in the number of packets per burst.

#### 4.1.4. Estimate Buffer Size

From the destination capture we can observe that the first burst of dropped packets corresponds to* Buffer *2 despite having a lowest filling rate than* Buffer *1. This is due to the time to completely fill* Buffer *2 is smaller than* Buffer *1, so size_*Buffer*_2__ < size_*Buffer*_1__.

**Figure 11:**
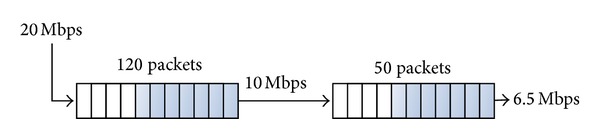
Estimated parameters for two concatenated buffers.

We obtained the buffer size for* Buffer *1 (switch) and* Buffer *2 (access point) using (5) with *n*_1_ = 240 and *n*_2_ = 143. The maximum buffer sizes are roughly 120 packets for the switch and 50 packets (average) for the access point, which correspond to the technical characteristics provided by the manufacturer. The test was repeated with different packet sizes obtaining the same results for buffer sizes, so we conclude that both buffers are measured in number of packets (Figure 11). This methodology can be used for a deep analysis of a single device, the most restrictive, for example, observing the packet size effect.

## 5. Conclusion

This paper has presented a methodology which is useful in order to describe the technical and functional characteristics of commercial buffers on a network path. This characterization is important, taking into account that the buffer may modify the traffic characteristics.

Tests using commercial devices have been deployed in a controlled laboratory scenario, including wired and wireless devices. Accurate results of the buffer size and other parameters have been obtained. We have demonstrated that buffers may be analyzed independently of other devices. As a future line, more than two buffers will be studied by packet loss pattern analysis.
